# Silicon-based photonic crystals fabricated using proton beam writing combined with electrochemical etching method

**DOI:** 10.1186/1556-276X-7-416

**Published:** 2012-07-23

**Authors:** Zhiya Dang, Mark BH Breese, Gonzalo Recio-Sánchez, Sara Azimi, Jiao Song, Haidong Liang, Agnieszka Banas, Vicente Torres-Costa, Raúl José Martín-Palma

**Affiliations:** 1 Centre For Ion Beam Applications (CIBA), Department Of Physics, National University Of Singapore, Singapore, 117542, Singapore; 2 Singapore Synchrotron Light Source (SSLS), National University of Singapore, 5 Research Link, Singapore, 117603, Singapore; 3 Departamento De Física Aplicada, Universidad Autónoma De Madrid, Cantoblanco, Madrid, 28049, Spain

**Keywords:** Proton beam writing, Defect density, Photonic band structure

## Abstract

A method for fabrication of three-dimensional (3D) silicon nanostructures based on selective formation of porous silicon using ion beam irradiation of bulk p-type silicon followed by electrochemical etching is shown. It opens a route towards the fabrication of two-dimensional (2D) and 3D silicon-based photonic crystals with high flexibility and industrial compatibility. In this work, we present the fabrication of 2D photonic lattice and photonic slab structures and propose a process for the fabrication of 3D woodpile photonic crystals based on this approach. Simulated results of photonic band structures for the fabricated 2D photonic crystals show the presence of TE or TM gap in mid-infrared range.

## Background

Periodically modulated refractive-index structures, i.e., a photonic crystal which can modulate the flow of electromagnetic waves, exhibit photonic band gaps under certain conditions [1,2]. As electron mobility in a semiconductor can be controlled by engineering the electronic bands of these materials, electromagnetic wave propagation inside a photonic crystal may be manipulated by machining its photonic bands [3]. Silicon-based photonic crystals are one of the most promising choices due to their easy integration in silicon technology, allowing applications in several fields, such as optical devices, including waveguides [4,5], resonators [6], etc. A lot of work has been reported on the fabrication and theoretical study of two-dimensional (2D) silicon-based photonic crystals [7] because of the advantages of easy integration and applications in planar platforms [8], such as planar waveguides [9,10]. Porous silicon-based photonic crystal is another promising candidate to be integrated in silicon technology [7,8,11]. To completely manipulate the flow of electromagnetic waves, a three-dimensional (3D) photonic crystal with complete band gap is required. Many methods have been reported to be able to fabricate 3D silicon-based photonic crystals with 3D complete band gap, such as double-angled reactive ion etching [12], macropore formation in silicon [13], glancing-angle deposition [14], and colloidal self-assembly [15]. One type of 3D photonic crystal that has attracted great attention is the 3D woodpile structure. Several techniques have been reported on the fabrication of 3D silicon-based woodpile structures, such as silicon double-inverse method [16] and layer-by-layer approach [17]. However, in the year 2000, Chow et al. reported the fabrication of a 2D photonic crystal slab capable of fully controlling light in all three dimensions [18], where the periodic dielectric structure is in only two dimensions, and index guiding is used to confine light in the third one. Most of the reported silicon-based photonic slabs are based on silicon-on-insulator platform [4-6].

In the present work, a method to fabricate 2D/3D silicon-based photonic crystals that uses high-energy proton beam writing and subsequent electrochemical etching of p-type bulk silicon wafers is presented. This technique uses either the whole defect regions at high ion fluence to completely inhibit the etching process or localized high defect density at the end-of-range region of high-energy protons at moderate fluence for the fabrication of silicon structures within the bulk silicon at certain depths, based on selective formation of porous silicon in other regions during subsequent anodization. A finely focused, high-energy ion beam [19] is scanned over the silicon wafer surface. As the ion beam penetrates the silicon, the crystal lattice is damaged, producing additional defects, which reduces the localized hole density and hole current [20,21]. The defect density for light ions, with energies greater than about 50 keV, peaks close to the end of their range [22]. By pausing the focused beam of different energy for different amounts of time at different locations, any pattern of localized damage can be built up. The irradiated wafer is then electrochemically anodized in an electrolyte of Hydrofluoric acid(HF). At a high ion fluence, the irradiated regions completely inhibit the formation of porous silicon and remain as silicon, based on which, Teo et al. has reported that fabrication of a periodic array of sub-micron diameter pillars is potentially important for the fabrication of photonic crystals [23]. While at a moderate ion fluence, only the buried regions with high defect density inhibit the porous silicon formation process. Thus, as the sample is etched beyond the depth of the ion range, the structure starts to become undercut due to isotropic etching, producing a silicon core that is surrounded by porous silicon. Multiple-energy proton irradiation can be used to create localized defects at different depths within the silicon wafer to fabricate multilevel 3D structures [24]. By varying the proton energy, the penetration depth changes, and subsequent etch steps enable the fabrication of true 3D silicon freestanding structures.

Additionally in this work, some sample structures of 2D photonic crystals are shown: a square lattice of silicon pillars in an air matrix, which utilize the complete inhibition of etching in irradiated regions, and a 2D photonic slab of air holes in silicon matrix, which utilizes the highly damaged regions at the end of range of ions in silicon. Theoretical photonic band structures of these structures were calculated, showing a complete transverse magnetic (TM) gap for the first structure and several complete transverse electric (TE) gaps for the second one. To further explore the fabrication of 3D photonic crystal structure using this approach, the fabrication of 3D silicon-based woodpile structure is proposed, and its initial result is shown.

## Methods

A nuclear microprobe at the Centre for Ion Beam Applications, National University of Singapore, was used [25]. High-energy protons with 200 keV to 1 MeV can be focused down to 100 nm. By controlling the duration time of protons on different points of the surface of a 0.02- or 0.4-Ω·cm p-type silicon wafer, designed defect distributions in the silicon wafers were built. Etching for irradiated silicon wafers was performed in an electrolyte of HF (48%): ethanol in the ratio of 1:1, with a current density of 40 mA/cm^2^ for 0.02-Ω·cm and 60 mA/cm^2^ for 0.4-Ω·cm wafers. A further step of dipping in KOH solution removed the porous silicon.

### 2D photonic lattice on silicon substrate: silicon pillars with square lattice

**Figure 1:**
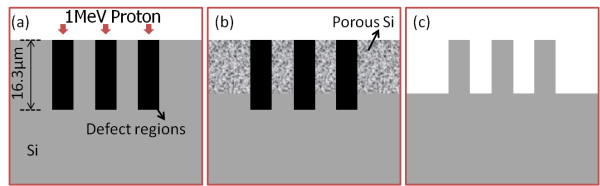
**Schematic cross-section view of 2D photonic lattice fabrication on silicon substrate. **(**a**) Proton beam-writing process and resultant defect distribution. (**b**) Selective formation of porous silicon in subsequent electrochemical etching in HF electrolyte. (**c**) Removal of porous silicon in KOH solution.

At high ion fluences, which are 1 × 10^17^/cm^2^ for 0.02-Ω·cm, and 1 × 10^16^/cm^2^ for 0.4-Ω·cm wafers, the defect density along the irradiation regions from the top surface to the end-of-range region is high enough to completely inhibit the electrochemical etching processes with the above etching conditions. As shown in Figure 1a, irradiation of 1-MeV protons with a high ion fluence in bulk silicon results in defect regions with high-enough density along the full range of 16.3 μm, which protons can penetrate. During electrochemical etching process, porous silicon is selectively formed in the nonirradiated regions, as shown in Figure 1b. The etching time for different current densities and wafer resistivity was well controlled to keep the etching depth less than 16.3 μm. After removal of porous silicon in KOH solution, silicon structures on the substrate were obtained, as in Figure 1c.

**Figure 2:**
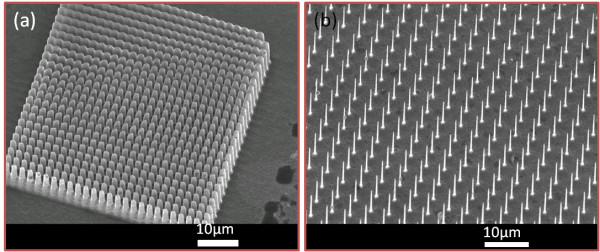
**Titled scanning electron microscope images of silicon pillars in square lattice. **(**a**) Period of 2 μm with a large *r*/*a* ratio, where *r* is the radius of the pillars and *a* is the lattice period. (**b**) Period of 4 μm with a small *r*/*a* ratio.

Patterns with square lattice of silicon pillars were designed with different periods for proton beam writing. Figure 2a shows a scanning electron microscope (SEM) image at 25° tilt. A 0.4-Ω·cm wafer was irradiated with a square lattice pattern with a 2-μm period using 1-MeV protons, which were focused to 400 nm in both directions, with fluence 5 × 10^16^/cm^2^. After etching with 60 mA/cm^2^ for 5 min and removing porous silicon with a KOH solution, silicon pillars in Figure 2a were obtained. In Figure 2b, a 0.4-Ω·cm wafer was irradiated with a square lattice with a larger period of 4 μm and a smaller beam size of 200 nm, with the same fluence, and etched for 6 min at the same current density.

### 2D freestanding photonic slab: a photonic slab with square lattice of air hole in silicon matrix

**Figure 3:**
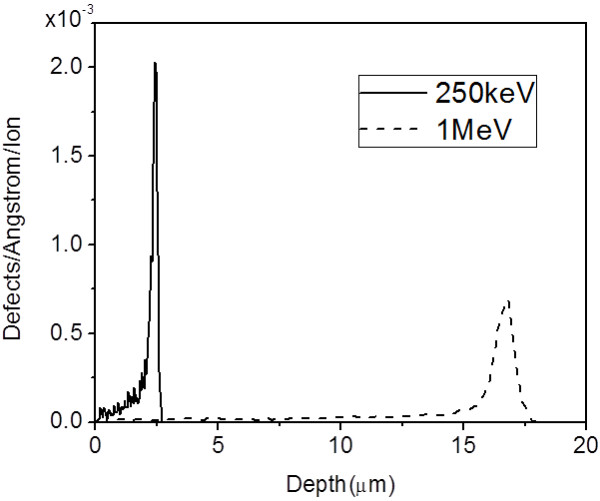
**Defect distribution from SRIM calculation.** Defect density distribution along the trajectory of ions for 250-keV and 1-MeV protons in silicon.

Figure 3 shows the defect density distribution versus depth for 250-keV and 1-MeV protons in silicon from Stopping and Ranges of Ions in Matter (SRIM) simulation [22]. Most of the defects concentrate at the end-of-range regions where the ions stop. At a moderate fluence for 250-keV protons, regions with high-enough defect density to inhibit formation of porous silicon are only located at a depth around 2.4 μm; thus, buried silicon wires form surrounded by porous silicon.

**Figure 4:**
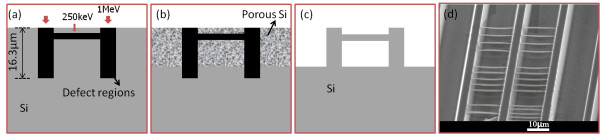
**Schematic of fabrication of freestanding silicon wires. **(**a**) Proton beam-writing process and resultant defect distribution in cross-section view. (**b**) Selective formation of porous silicon in subsequent electrochemical etching. (**c**) Removal of porous silicon in KOH solution. (**d**) SEM image of freestanding silicon wires with three different spacings.

To obtain freestanding structures, a high-energy proton beam of 1 MeV, which has a deep penetration depth in silicon, was used to irradiate lines with an extremely high fluence, 1 × 10^12^/cm, at the same area which function as supports, as in Figure 4a. Line fluence is used here in the line irradiation case where the size of ion beam is smaller than the lateral width of high defect regions. In subsequent electrochemical etching, the etching time and current density for different resistivity wafers were carefully controlled to completely undercut the end-of-range regions of 250-keV protons, but not reach the end-of-range of 1-MeV protons, as in Figure 4b. Subsequent dipping in dilute KOH solution removed the porous silicon, and freestanding silicon wires supported by thick walls were obtained, as in Figure 4c. Figure 4d shows freestanding silicon wires with three different spacings, where 250-keV protons were focused to 100 nm and irradiated with a line fluence of 1 × 10^11^/cm. Based on this, fabrication of a 2D photonic slab of a square lattice of air holes in a silicon matrix was designed.

**Figure 5:**
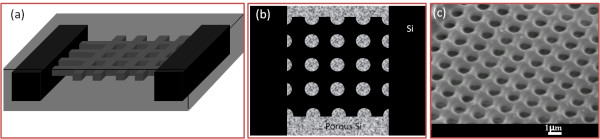
**Fabrication of 2D photonic slab with square lattice of air hole in a silicon matrix. **(**a**) Proton beam-writing process and resultant 3D defect distribution. (**b**) Selective formation of porous silicon in circular regions in subsequent electrochemical etching in HF electrolyte. (**c**) SEM image of the freestanding photonic slab structure after removal of porous silicon.

Two sets of lines were irradiated horizontally and vertically in the same area using 250-keV protons on 0.02-Ω·cm silicon wafers to create defect distribution, as shown in Figure 5a, in which the defect density at the intersecting parts of the lines is doubled. Thus, in the etching process, the surrounding area of the intersecting part is not etched as well, giving rise to the formation of porous silicon in circular regions, as shown in Figure 5b. After 4 min of etching with a current density of 40 mA/cm^2^ and a subsequent removal of porous silicon, a freestanding 2D photonic slab with a square lattice of air hole in silicon matrix was obtained, as shown in Figure 5c. This structure was fabricated with an ion fluence of 8 × 10^10^/cm with a period of 1.5 μm.

### 3D woodpile structure

**Figure 6:**
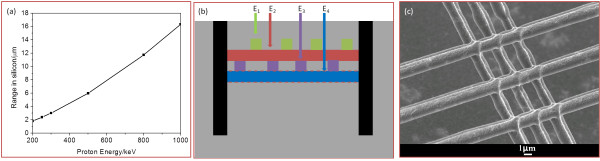
**Fabrication of 3D woodpile structures. **(**a**) Range of protons in silicon as a function of proton energy from SRIM calculation. (**b**) Regions with high defect density at four different depths using four energies with accurate alignment (dashed line demonstrates the silicon wires of the fourth layer is located half of period with respect to the second layer). (**c**) SEM image of the initial result on a two-layer structure.

By tuning the energy of ions, end-of-range regions with high defect density at moderate ion fluence can be generated at different depths in the wafer, and after a subsequent electrochemical anodization, silicon wires at different depths of the silicon wafer were obtained. SRIM [22] calculations in Figure 6a show that the range of protons in silicon varies from 1.8 to 16.3 μm when the proton energy increases from 200 keV to 1 MeV.

Here, we propose the fabrication of a 3D woodpile structure using proton beam writing to generate end-of-range regions with high defect density at different depths. In order to fabricate woodpile structure with one period, protons with four different energies, E_1_, E_2_, E_3_, and E_4_, will be required to irradiate lines in the same area with suitable fluence and alignment, as shown in Figure 6b. Figure 6c shows an initial result on two-layer freestanding silicon wires in two directions using 250 and 200-keV protons, respectively, in 0.02-Ω·cm silicon wafers supported by thick walls from 1-MeV protons. By carefully controlling the alignment, ion energy, ion fluence, and etching current density, a 3D woodpile structure with complete band gap lying in the mid-infrared (IR) range should be possible.

## Results and discussions

The MIT photonic band package [26] has been used to study the photonic band structure (PBS) of these photonic crystal structures. The software is based on conjugate-gradient minimization of the Rayleigh quotient in a plane-wave basis [27].

### PBS for silicon pillars with square lattice in an air matrix on silicon substrates

**Figure 7:**
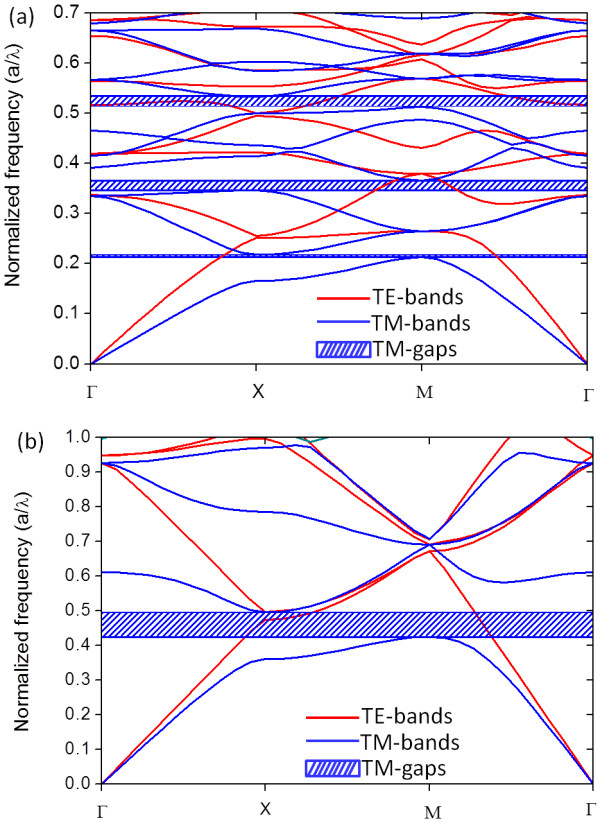
**Photonic band structures of square lattice of Si pillars. **(**a**) 2D PBS of the structure shown in Figure 2a. (**b**) 2D PBS of the structure shown in Figure 2b.

Figure 7a,b shows the computed 2D PBS for the experimental structures shown in Figure 2a,b, respectively. In both figures, a TM photonic gap opens between the first and second band. However, the gap size is much higher in Figure 7b. This is due to the different concentration factors in both structures. In Figure 7b, the radius of the Si pillars is *r* = 0.1*a*, where *a* is the lattice period, and the gap opens from 0.422 to 0.495 of the normalized frequency. While the radius of the Si pillar in the structure of Figure 7a is *r* = 0.415*a*, the gap opens from 0.210 to 0.218 of the normalized frequency. As the first photonic band concentrates its energy in the high-dielectric-constant region, i.e., in the Si pillar, the second band concentrates its energy in the low-dielectric-constant region in order to be orthogonal to the first band [3]. When the ratio *r*/*a* is smaller, the different concentration factors between both bands increase, and the gap size is higher. If the radius of the Si pillar is too small, the first band cannot concentrate its energy in them, and the gap disappears.

On the other hand, in Figure 7a, other gaps open at higher frequencies. Between the third and fourth bands, a gap opens from 0.345 to 0.365 of the normalized frequency, and between the sixth and seventh, from 0.513 to 535. However, no TE gap opens in either structure. The flexibility of the fabrication process allows varying the radius of the Si pillar and the lattice period to tune the frequency ranges where the gaps can be opened for a specific application.

### PBS for photonic slab with square lattice of air hole in silicon matrix

In this case, as a freestanding structure was studied, novel approaches were needed. First, the eigenstates of the slab were calculated using a z-supercell approach, where guided bands were unaffected. Then, the light cone was obtained and overlapped [28].

**Figure 8:**
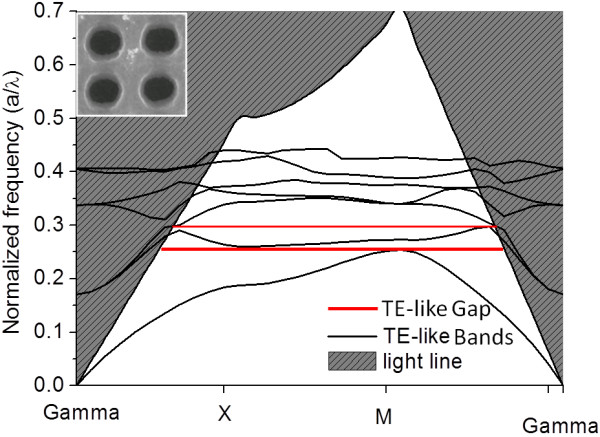
**Photonic band structure for TE-like modes for the structure in Figure**5**(c).** 2D photonic slab with square lattice of air hole in silicon matrix.

Figure 8 shows PBS for TE-like modes; bands with even symmetry respect reflections through a *z* plane (*z* directions being the height slab direction) for the photonic slab of air holes with square lattice in silicon matrix shown in Figure 5c, with *r*/*a* = 0.3125 and *h*/*a* = 0.75, where *r* is the radius of the air hole, *h* is the thickness of the slab, and *a* is the period of the lattice.

**Figure 9:**
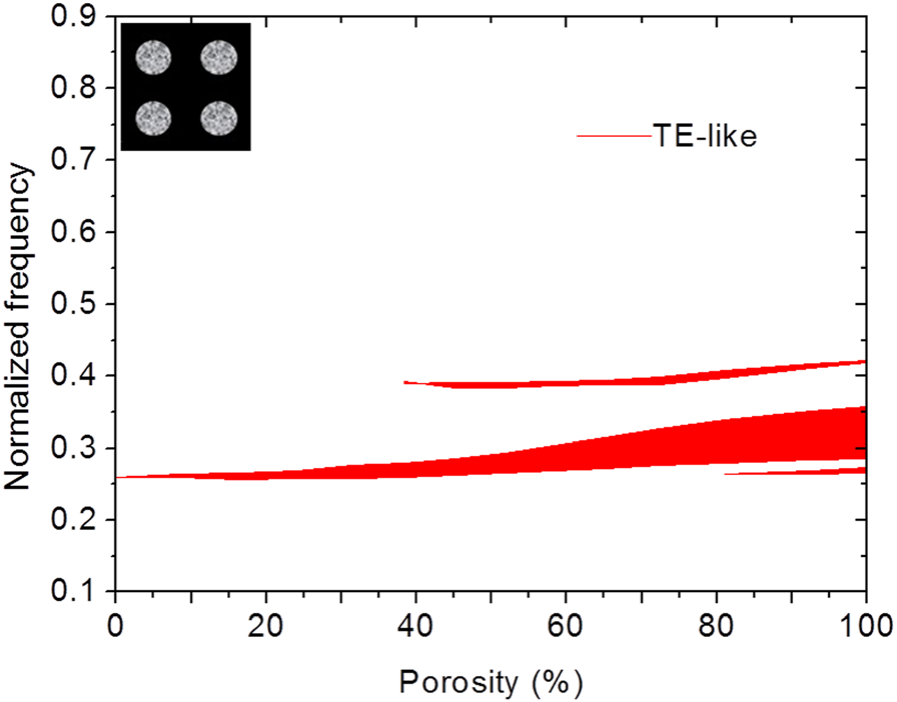
**Gap map of photonic slab of square lattice of porous silicon hole in silicon matrix.** With *r*/*a* = 0.3125 and *h*/*a* = 0.75.

It shows two gaps around normalized frequency *a*/λ = 0.26 and 0.29, where *a* = 1.5 μm. PBS for TM-like modes shows no gap. The frequency range and gap size can be tuned and optimized by varying irradiation and etching conditions. Photonic crystal structures based on this approach of selective formation of porous silicon have an extra degree of tuning from porous silicon, where removal process of porous silicon in KOH etching is not conducted instead. Figure 9 shows the gap map of porosity for a photonic slab of square lattice of porous silicon hole in silicon matrix, with *r*/*a* = 0.3125 and *h*/*a* = 0.75, where *r* is the radius of the porous silicon hole, *h* is the thickness of slab, and *a* is the period of lattice.

## Conclusions

We have shown a highly flexible approach of using electrochemical etching following proton beam writing of bulk p-type silicon wafers to fabricate 2D/3D silicon-based photonic crystals. Simulation studies show that the structure of silicon pillars with square lattice has TM gaps in the mid-IR range, while the structure of air holes with square lattice in silicon matrix has TE gaps in the same wavelength range. Photonic crystals fabricated using this approach have an extra degree of tuning from porous silicon. Based on the platform of bulk wafers and being compatible with silicon technology, this flexible fabrication method is a promising candidate for the development of silicon-based photonic crystals.

## Competing interests

The authors declare that they have no competing interests.

## Authors’ contributions

ZD carried out the fabrication process. GRS, VTC and RJMP carried out the simulation study. MB and ZD conceived of and designed the 2D photonic structures and 3D woodpile structure. ZD, MB, SA, JS, HL, and AB discussed the results obtained from experiments. ZD wrote the manuscript, and GR and MB helped draft the manuscript. All authors read and approved the final manuscript.

## References

[B1] Yablonovitch E Inhibited spontaneous emission in solid-state physics and electronics Phys Rev Lett 1987 58 2059 2062 10.1103/PhysRevLett.58.2059 10034639

[B2] Krauss TF Rue RMDL Brand S Two-dimensional photonic-bandgap structures operating at near-infrared wavelengths Nature 1996 383 699 702 10.1038/383699a0

[B3] Joannopoulos JD Photonic Crystals: Molding the Flow of Light 2008 Princeton University Press, Princeton

[B4] Loncar M Doll T Vuckovic J Scherer A Design and fabrication of silicon photonic crystal optical waveguides J Light Technol 2000 18 1402 1411

[B5] Moll N Vlasov YA McNab SJ Broad bandwidth double-trench waveguides in silicon-on-insulator photonic crystal slabs Proc SPIE 2004 5360 145

[B6] Chiu WY Huang TW Wu YH Chan YJ Hou CH Chien HT Chen CC A photonic crystal ring resonator formed by SOI nano-rods Opt Express 2007 15 15500 15506 10.1364/OE.15.015500 19550835

[B7] Martín-Palma RJ Torres-Costa V Manso M Martínez-Duart JM Finite-thickness photonic crystals based on nanostructured porous silicon for optical sensing Journal of Nanophotonics 2009 3 031504 10.1117/1.3079805

[B8] Martín-Palma R Manso M Arroyo-Hernández M Torres-Costa V Martínez-Duart J Nanostructured-porous-silicon-based two-dimensional photonic crystals Appl Phys Lett 2006 89 053126 10.1063/1.2335586

[B9] Søndergaard T Lavrinenko A Large-bandwidth planar photonic crystal waveguides Opt Commun 2002 203 263 270 10.1016/S0030-4018(02)01172-0

[B10] Borel PI Frandsen LH Leon JB Niemi T Lavrinenko AV Optimization and applications of planar silicon-based photonic crystal devices Proc SPIE 2005 601410 1 601410.9

[B11] Recio-Sánchez G Torres-Costa V Manso-Silván M Martín-Palma RJ Nanostructured porous silicon photonic crystal for applications in the infrared Journal of Nanotechnology 2012

[B12] Takahashi S Suzuki K Okano M Imada M Nakamori T Ota Y Ishizaki K Noda S Direct creation of three-dimensional photonic crystals by a top-down approach Nat Mater 2009 8 721 725 10.1038/nmat2507 19668208

[B13] Matthias S Müller F Gösele U Simple cubic three-dimensional photonic crystals based on macroporous silicon and anisotropic posttreatment J Appl Phys 2005 98 023524 10.1063/1.1993752

[B14] Kennedy SR Brett MJ Toader O John S Fabrication of tetragonal square spiral photonic crystals Nano Lett 2002 2 59 62 10.1021/nl015635q

[B15] Ozin GA Yang S The race for the photonic chip: colloidal crystal assembly in silicon wafers Adv Funct Mater 2001 11 95 104 10.1002/1616-3028(200104)11:2<95::AID-ADFM95>3.0.CO;2-O

[B16] Staude I Thiel M Essig S Wolff C Busch K Von Freymann G Wegener M Fabrication and characterization of silicon woodpile photonic crystals with a complete bandgap at telecom wavelengths Opt Lett 2010 35 1094 1096 10.1364/OL.35.001094 20364228

[B17] Wang L Zhang S Wang Q Chen J Jiang W Chen RT Fabrication of three-dimensional (3D) woodpile structure photonic crystal with layer by layer e-beam lithography Appl Phys A 2009 95 329 334 10.1007/s00339-009-5076-7

[B18] Chow E Lin S Johnson S Villeneuve P Joannopoulos J Wendt JR Vawter GA Zubrzycki W Hou H Alleman A Three-dimensional control of light in a two-dimensional photonic crystal slab Nature 2000 407 983 986 10.1038/35039583 11069173

[B19] Breese MBH Jamieson DN King PJC Materials Analysis Using a Nuclear Microprobe 1996 John Wiley & Sons Ltd, Sussex 428

[B20] Svensson B Mohadjeri B Hallén A Svensson J Corbett J Divacancy acceptor levels in ion-irradiated silicon Physical Review B 1991 43 2292 10.1103/PhysRevB.43.2292 9997504

[B21] Breese M Champeaux F Teo E Bettiol A Blackwood D Hole transport through proton-irradiated p-type silicon wafers during electrochemical anodization Physical Review B 2006 73 035428

[B22] Ziegler JF Ziegler M Biersack J SRIM–The stopping and range of ions in matter (2010) Nucl Instrum Methods Phys Res, Sect B 2010 268 1818 1823 10.1016/j.nimb.2010.02.091

[B23] Teo E Breese M Tavernier E Bettiol A Watt F Liu M Blackwood D Three-dimensional microfabrication in bulk silicon using high-energy protons Appl Phys Lett 2004 84 3202 3204 10.1063/1.1723703

[B24] Azimi S Breese M Dang Z Yan Y Ow Y Bettiol A Fabrication of complex curved three-dimensional silicon microstructures using ion irradiation J Micromech Microengineering 2012 22 015015 10.1088/0960-1317/22/1/015015

[B25] van Kan J Bettiol A Ansari K Shao P Watt F Proceedings of the 17th IEEE International Conference on Micro Electro Mechanical Systems: 25–29 January 2004 Improvement in proton beam writing at the nano scale 2004 IEEE Xplore, Maastricht. New York 673 676

[B26] Johnson SG Joannopoulos JD Block-iterative frequency-domain methods for Maxwell’s equations in a planewave basis Opt Express 2001 8 173 190 10.1364/OE.8.000173 19417802

[B27] Sailor W Mueller F Villeneuve PR Augmented-plane-wave method for photonic band-gap materials Physical Review B 1998 57 8819 8822 10.1103/PhysRevB.57.8819

[B28] Johnson SG Fan S Villeneuve PR Joannopoulos J Kolodziejski L Guided modes in photonic crystal slabs Physical Review B 1999 60 5751 10.1103/PhysRevB.60.5751

